# Three-dimensional shape measurement for the steep surface using DMD camera

**DOI:** 10.1038/s41598-022-24942-8

**Published:** 2022-12-06

**Authors:** Shoubo Zhao, Yuqiang Yang

**Affiliations:** 1 School of Mechanical Engineering, Guangdong Ocean University, Zhanjiang, 524088 China; 2 Guangdong Provincial Engineering Research Center for Ship Intelligence and Safety, Guangdong Ocean University, Zhanjiang, 524088 China

**Keywords:** Applied optics, Imaging and sensing, Mechanical engineering

## Abstract

Three-Dimensional shape measurement has been confronted with the ambiguity on the steep surface. To address the problem, compressed sensing theory is employed to reconstruct phase shifting images in DMD camera. Specially, every CCD pixel in the region of interest is aligned to *N* × *N* DMD mirrors to construct DMD camera. The one-dimensional measurement matrices are chosen to collect the measured values on CCD pixel according to the directional judgement of original gradient image. Due to the enhancement of the spatial sampling frequency and the noise robustness, the reconstructed sinusoidal stripe images are utilized to obtain the three-dimensional model of the steep surface accurately. We measure the planes with various slopes to discuss the measurement capability. The comparative experiments show that our proposed method can correct the deformed phase and repair the defect on the steep surface.

## Introduction

Spatial sampling frequency is the key factor in three-Dimensional shape measurement, which is limited by spatial resolution of sensor. Once the image resolution and the parameters of objective lens are fixed in traditional imaging technology, it is no way to enhance spatial sampling frequency of traditional imaging system. However, partial slope factor of complex surface is often beyond the capacity of spatial sample, which produces the sampling information loss in the region of steep surface. Take the involute gear for example, which is widely used in mechanical engineering. To ensure the stability of gear drive and the uniformity of load distribution, it is necessary to conduct three-Dimensional shape measurement to analyze geometric error such as pressure angle, thickness, addendum and dedendum. While 3D shape measurement is confronted with the challenge of the continuous increasement in the slope factor of the involutes profile. The limitation of spatial sampling frequency causes the unwrapped phase gap. The defect often manifests as these holes and fractures in the reconstruction surface. The same phenomenon occurs in other concave and convex parts such cam, hemispheroid and inner bore.

There are some traditional strategies to measure the steep surface. Many researchers make use of coded structured light to enhance sub-pixel accuracy^1–4^. Fang Ji proposed an adaptive Canny edge detection method with two phases to distinguish the light stripes accurately^5^. Kai Liu embedded a period cue into the projected pattern to solve the depth ambiguity which caused by increasing the number of pattern stripes^6^. Zhan Song designed a gridline pattern to improve decoding accuracy of 3D sensing system^7^. Rosario Porras-Aguilar optimized grey-level projection patterns with a minimal gradient to reduce phase error near the border of the stripe^8^. However, since the reflectance variation on measured surface produces the ambiguity of intensity pattern, the sub-pixel accuracy estimations for structured light in above coding technologies could not avoid random errors of the ambiguity in 3D measurement^9^. In addition, focus variation technology is also a common way of measuring steep surface. Reinhard Danzl exploited the small depth of focus of an optical system with vertical scanning to enhance the maximum measurable slope angle^10^. Lewis Newton analyzed the measurement process control parameters to improve the quality of the focus variation measurement results^11^. Yukitoshi proposed a phase-shifting method merging into focus variation technology to analyze the contrast distribution by projecting grating pattern^12^. However, since the performance of focus variation measurement is significantly affected by the control precision of instrument vertical scanning process, the vertical scanning error along the optical axis restricts the measurement capability for the steep Surface. Imaging from different angle is an effective method to void steep surface in 3D measurement^13–16^. Although we can bypass the problem by employing the multiple view imaging structure, it is necessary to tackle the problem head on by enhancing imaging resolution of the traditional imager. Some researchers proposed interpolation algorithms to improve the measurement accuracy by up-sampling original image^17–19^. Wenzhe proposed algorithm for the accurate measurement of 3D cardiac function by reconstructing a super-resolution image within an expectation maximization framework^17^. Yang presented an iterative refinement approach to up-sample the original low-resolution image^18^. Peter proposed a method for super-resolution 3D laser scanning by which the length of the line segments along the camera rays can be tightened^19^. We pursue a goal that imaging resolution is improved to enhance spatial sampling frequency on the steep surface more effectively.

Compressed sensing (CS) as a signal processing technology for under-sampling and reconstructing a signal has been widely applied on medical imaging, remote sensing and industrial inspection over the past decade, since the signal may be recovered with far fewer samples than the Nyquist–Shannon sampling theorem requires^20–24^. Marco in Rice University designed a single-pixel camera based on the spatial light modulator (SLM) with CS theory^25^. SLM is employed to provide the measurement matrices by being imposed the modulation pattern. The modulated light is then focused onto a single photon detector. Single photon detector integrates the inner product of measurement matrices and the measured object to yield a set of sampled values. The set of sampled values are used to recover the object image via minimization. As a result, the single-pixel camera can image the scene with higher spatial sampling frequency than the single photon detector^26^. An alternative advantage is that the single-pixel camera is robust in the presence of noise, since one or more measurements can be lost without corrupting the entire reconstruction in CS theory^27,28^. This paper are inspired by previous works on single-pixel imaging technology and CS theory^29^. We try to use DMD camera, which is merged with CS theory, to improve the three-Dimensional shape measurement for steep surface. DMD camera can process the spatial array of incident rays before image formation. This modulation improves the quality of phase-shifting fringe image by reshaping waveform. The compensation of phase error and the suppression of noise are beneficial to three-Dimensional shape measurement.

The paper is organized as follows: Sect. 2 describes DMD camera architecture and alignment method. The measurement matrices for reconstruction of the sinusoidal stripe images are designed to measure three-dimensional shape in Sect. 3. Subsequently, experiments for 3D measurement are implemented to verify the performance of our method in Sect. 4. Finally, this research is concluded to discuss the advantage and limitation in Sect. 5.

### DMD camera system

**Figure 1:**
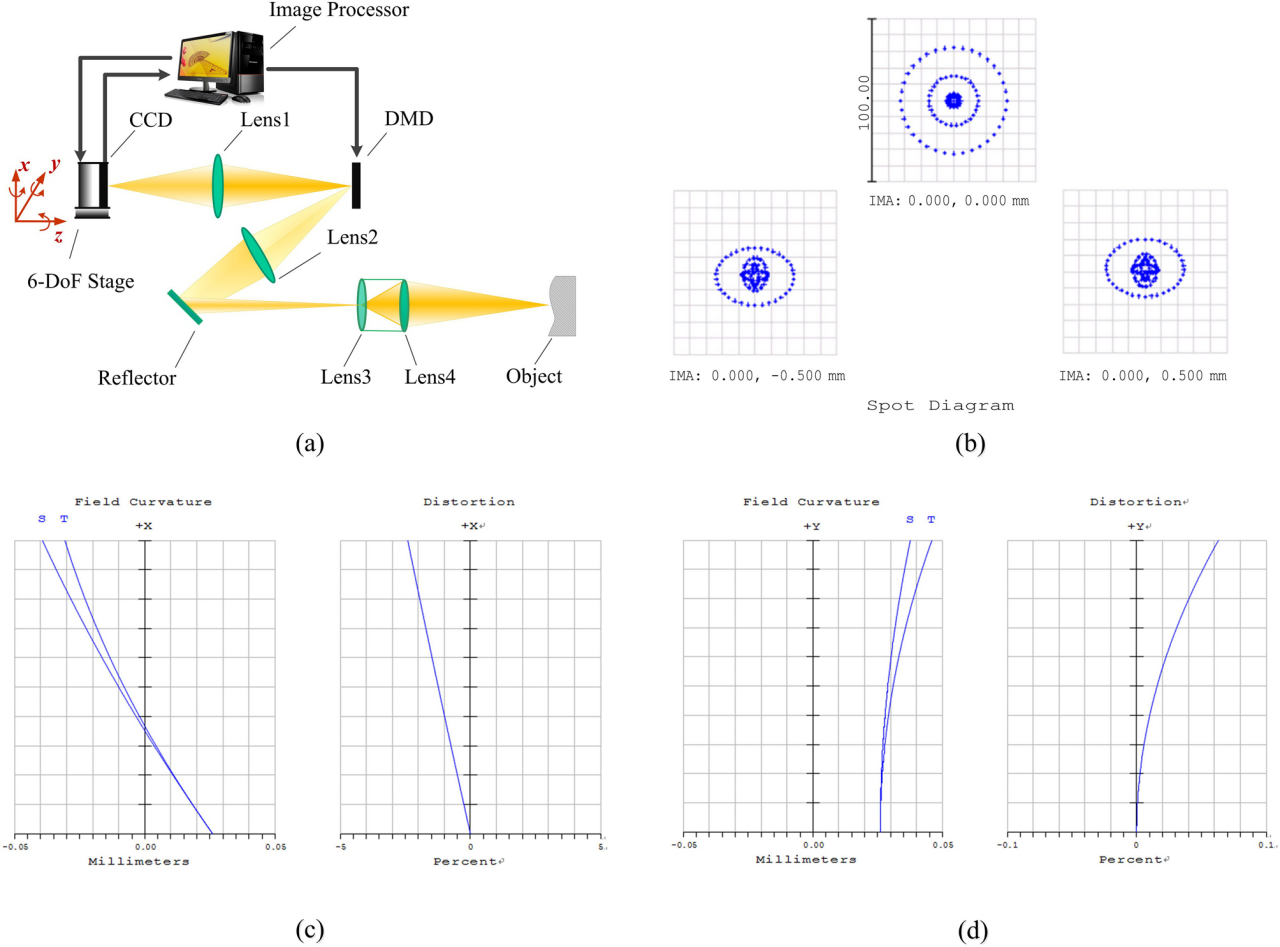
DMD imaging system. (**a**) The architecture of DMD camera. (**b**) The spot diagram of DMD camera system. (**c**) The distortions on x-axis. (d) The distortions on y-axis.

We constructed an imaging system called DMD camera that was applied on 3D shape measurement and noise removal^30,31^. The difference between DMD camera and single-pixel camera is mainly that the single photon detector is replaced by array charge coupled device (CCD). The schematic of DMD camera is shown in Fig. 1a. The system is composed of CCD, DMD, 6-DoF stage, image processor and four imaging lenses (Lens1, Lens2, Lens3 and Lens4). The pixel of CCD collects the reflected photons from the corresponding segment of DMD. 6-DoF assemble stage with piezoelectric actuators is conducted to adjust the angular and linear displacement of CCD according to the feedback from image processor. Zemax optics software is employed to analyze image quality. Figure 1b is the spot diagram of DMD camera system. The maximum radius of elliptical Airy spot is 1.894um. Imaging distortion on x-axis is inevitable due to Scheimpflug condition. Figure 1c,d are respectively the distortions on x-axis and y-axis. The distortion on x-axis is obviously bigger than the distortion on y-axis. And the linear growth is consistent with previous theoretical derivation in Ref^30^.

The paraxial magnification of Lens1 can be scanned from × 0.1 to × 10. Piezoelectric actuators (Thorlabs NanoMax320) are provided for fine adjustment with 20 nm resolution. DMD (Discovery 4100) provides 8bpp and a resolution of 1920 × 1080; DMD mirror element is 7.6 × 7.6 microns. CCD (Teli-BU30) allows for 8 bits per pixel (bpp) of precision in RAW mode and a resolution of 768 × 576; CCD pixel is 7.4 × 7.4 microns in size.

**Figure 2:**
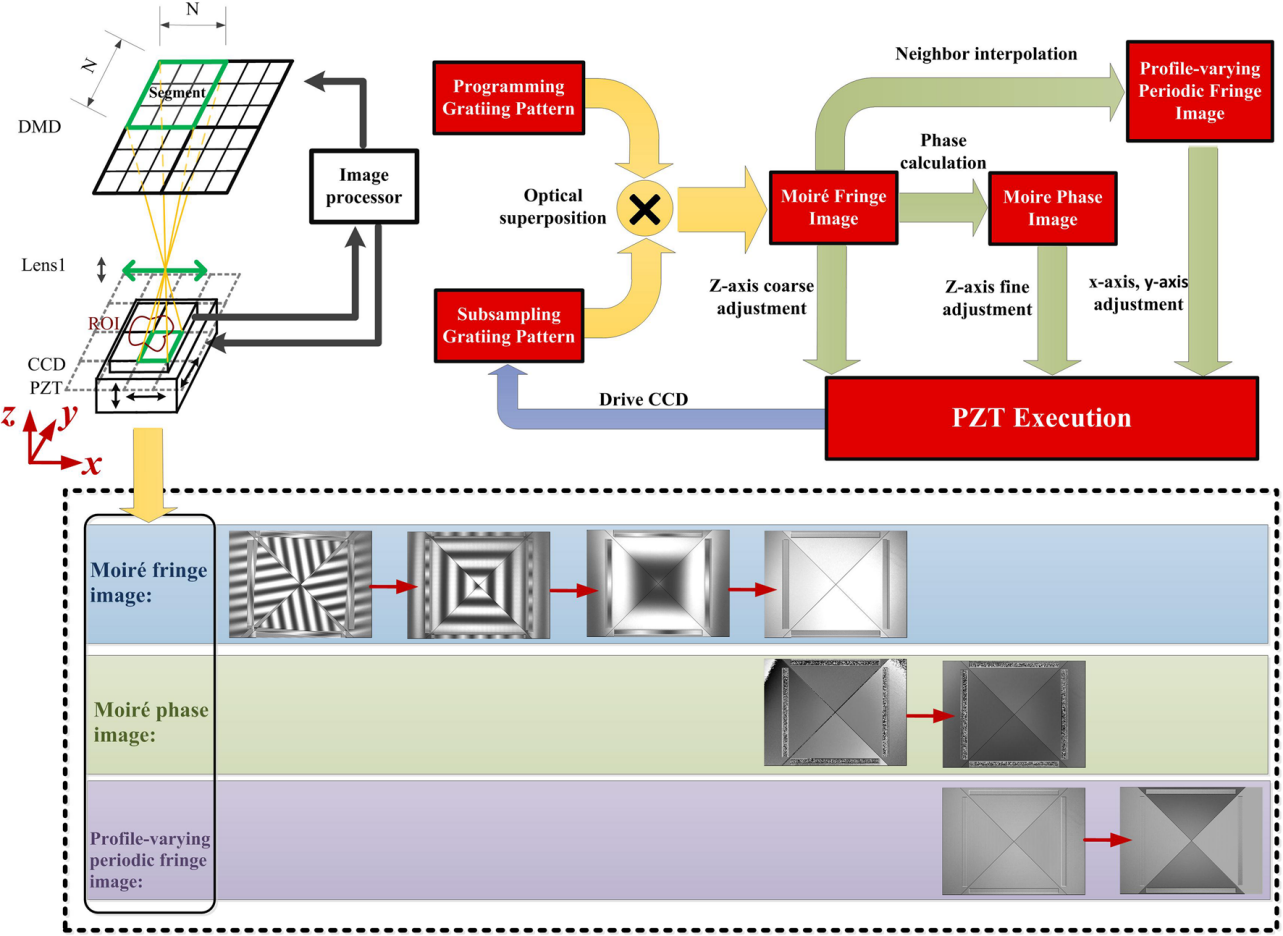
The closed-loop system for accurate alignment between CCD and DMD.

To utilize CS imaging technology, it is necessary that every CCD pixel in the region of interest (ROI) is assigned to multiple DMD mirrors. Consequently, DMD is divided into an array of segments, all of which contains *N* × *N* mirrors (*N* be integer). As shown in Fig. 2, a closed-loop system composed of piezoelectric actuators, CCD, Lens1, DMD and image processor is constructed to achieve accurate alignment between DMD and CCD, before DMD camera is on the working state. The alignment between DMD and CCD involves six degree of freedom (DoF) adjustments including three linear displacements on the horizontal axis x, the vertical axis y, the optical axis z and three angular displacements on pitch angle, yaw angle, roll angle. DMD can be regarded as one grating by programming the state of mirror array to form black and white line patterns. Likewise, CCD can be regarded as the alternative grating by using the subsampling technique. In other words, the pixels of the original image will be selected with the period of $f$, and unselected pixels will be interpolated by the neighbor selected pixel. The programming grating pattern is focused onto the subsampling grating pattern by Lens1 to product optical superposition phenomenon called moiré fringe. Moiré fringe image can be expressed as:

1 $$ I_{m}^{i} (x,y) = I\left( {f \cdot Floor(\frac{x}{f}) + i,f \cdot Floor(\frac{y}{f}) + i} \right) $$

where function $Floor[ \bullet ]$ is to get the integer towards minus infinity. $(x,y)$ is the CCD pixel coordinate. $I(x,y)$ is the original image captured by CCD. The symbol $f$ denotes the period of grating in CCD. Shifting phase in the $i{\text{th}}$ moiré image is $2i\pi /f$, $i$ = 1, 2, …, $f$. By observing the moiré fringe image, the roll, the pitch and the yaw angle are adjusted to obtain the intermediate state that the moiré fringe parallels the original grating fringe.

Moiré fringe image is used for phase calculation and neighbor interpolation in the image processor to obtain the moiré phase image and the profile-varying periodic fringe image respectively. The moiré phase image can be described by:

2 $$ \varphi (x,y) = \arctan \left( {\frac{{\sum\nolimits_{i = 1}^{f} {I_{m}^{i} \cdot \sin (2i\pi /f)} }}{{\sum\nolimits_{i = 1}^{f} {I_{m}^{i} \cdot \cos (2i\pi /f)} }}} \right) $$

By observing the moiré phase image, the paraxial magnification and z displacement along the optical axis are adjusted to make moiré phase distribution uniform. At this state, the frequency difference between the CCD and DMD equals to zero.

The neighbor interpolation can be described by:

3 $$ I_{s} (x,y) = I\left( {\frac{f}{2N} \cdot Floor(\frac{2Nx}{f}) + Mod(2Nx,f),} \right.\left. {\frac{f}{2N} \cdot Floor(\frac{2Ny}{f}) + Mod(2Ny,f)} \right) $$

where function $Mod(divident,divisor)$ is to get the remainder. The symbol $N*N$ is the mirror number in the segment of DMD. By observing the subsampling image, x-axis and y-axis displacements are adjusted to eliminate the initial phases of moiré fringe. Image processor can calculate the required displacements in 3-axis directions of PZT. PZT receives the feedback and execute the 3-axis adjustments until accurate alignment between the segment of DMD and the pixel of CCD is completed. The accurate alignment approach using the four-step procedure was discussed in detail in Ref^32^.

## Method

**Figure 3:**
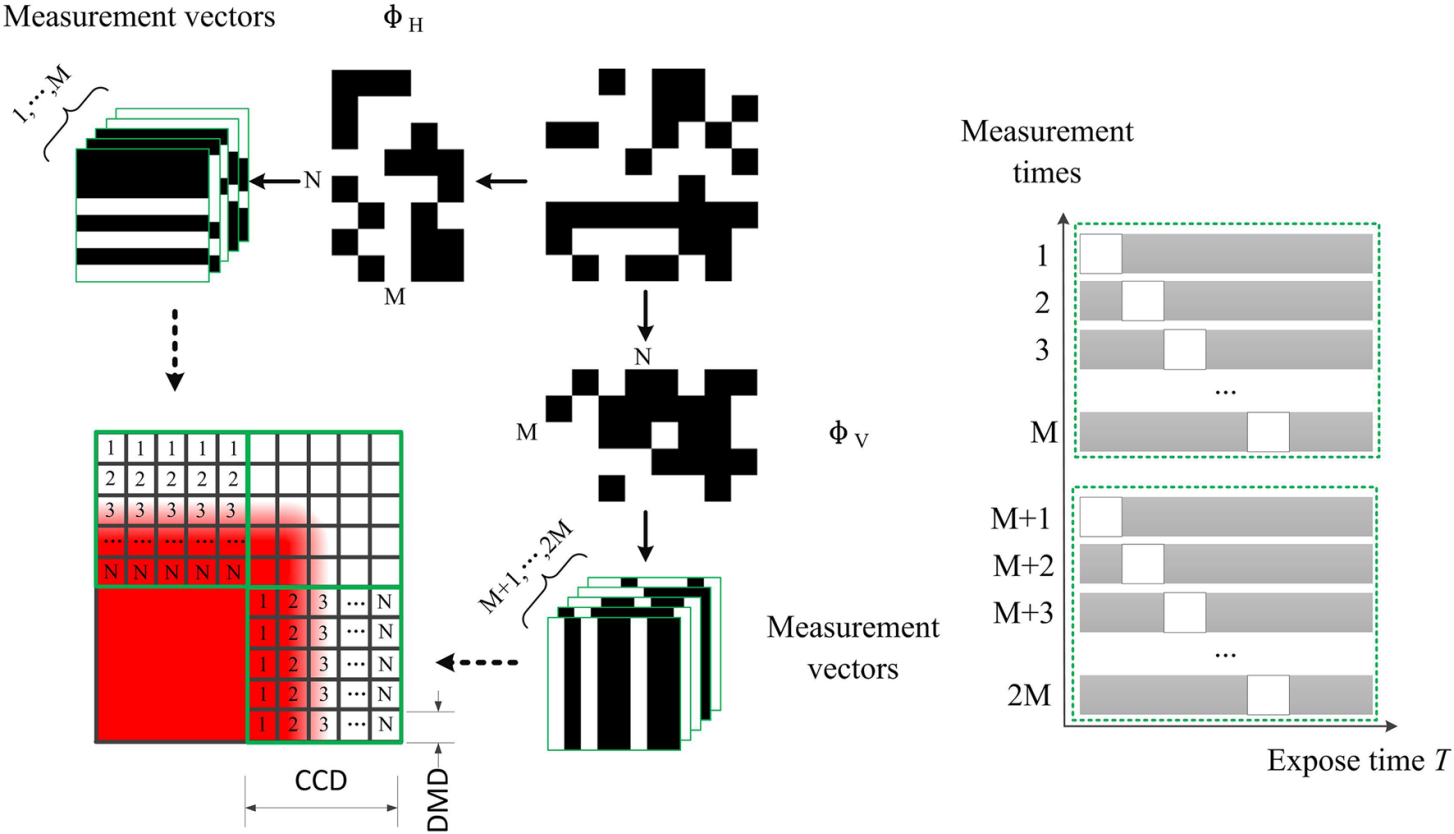
The CS-based imaging schematic of DMD camera.

As an indispensable factor of CS theory, measurement matrix is employed to acquire the under-sampling image. Figure 3 is the procedure of image acquisition in DMD camera. We first choose an orthogonal square matrix with uniform distribution such as Hadamard matrix, Bernoulli random matrix and Gaussian random matrix to ensure the restricted isometry property (RIP). The orientation of measurement vector is determined by comparing the horizontal gradient $g_{x}$ and vertical gradient $g_{y}$ of CCD pixel in original object image. If the measurement vector is x orientation ($g_{x} (x,y) < g_{y} (x,y)$), measurement matrix $\Phi_{H}$ is obtained by randomly selecting *M* columns in the orthogonal square matrix. Alternatively, if the measurement vector is y orientation ($g_{x} (x,y) > g_{y} (x,y)$), measurement matrix $\Phi_{V}$ is obtained by randomly selecting *M* rows in the orthogonal square matrix. The DMD pattern sequence is represented by

4 $$ DMD_{m} (u,v) = \left\{ {\begin{array}{*{20}l} {\Phi_{H} \left( {m,\bmod (v,N)} \right)\begin{array}{*{20}l} {} & {g_{x} \left( {\bmod (u,N),\bmod (v,N)} \right) < g_{y} \left( {\bmod (u,N),\bmod (v,N)} \right)} \\ \end{array} } \\ {\Phi_{V} \left( {\bmod (u,N),m} \right)\begin{array}{*{20}l} {} & {g_{x} \left( {\bmod (u,N),\bmod (v,N)} \right) > g_{y} \left( {\bmod (u,N),\bmod (v,N)} \right)} \\ \end{array} } \\ \end{array} } \right. $$

where $(u,v)$ is the DMD mirror coordinate. The symbol $m$ denotes the ordinal number of the DMD pattern, $m = 1,2,3 \ldots {\text{M}}$. CCD pixel collects the measured value $Y_{x,y} (m)$ when DMD provides the $m$ th pattern, i.e., DMD camera captures *M* images after *M* times exposure. Assuming that $I_{x,y} (n)$ is the original scene signal of arbitrary pixel (x, y), the measured values $Y_{x,y} (m)$ can be expressed by

5 $$ Y_{x,y} (m) = \Phi_{H}^{T} I_{x,y} (n)\quad {\text{or}}\quad Y_{x,y} (m) = \Phi_{V} I_{x,y} (n) $$

where $\Phi_{H}^{T}$ is the transpose of the matrix $\Phi_{H}$. Since 3D measurement is implemented by projecting the structure light pattern with sinusoidal stripes in this research, discrete cosine transform basis is suitable for the sparse representation of the stripe image. Discrete cosine transform matrix $\Psi$ is size $N \times N$. The mirror number in the DMD segment is equal to the size of discrete cosine transform matrix $\Psi$. To reconstruct the original scene signal $I_{x,y} (n)$, orthogonal matching pursuit (OMP) algorithm is utilized to solve the optimization problem of the L0-norm^33^.

6 $$ \overset{\lower0.5em\hbox{$\smash{\scriptscriptstyle\frown}$}}{I}_{x,y} = \arg \min \left\| {I_{x,y} } \right\|_{0} \quad {\text{s.t}}\quad \left\| {Y_{x,y} - \Phi \Psi \overset{\lower0.5em\hbox{$\smash{\scriptscriptstyle\frown}$}}{I}_{x,y} } \right\|_{2} < \varepsilon $$

where the optimal solution $$\overset{\lower0.5em\hbox{$\smash{\scriptscriptstyle\frown}$}}{I}_{x,y}$$ is the reconstructed vector of the original scene signal $$I_{x,y} (n)$$.

**Figure 4:**
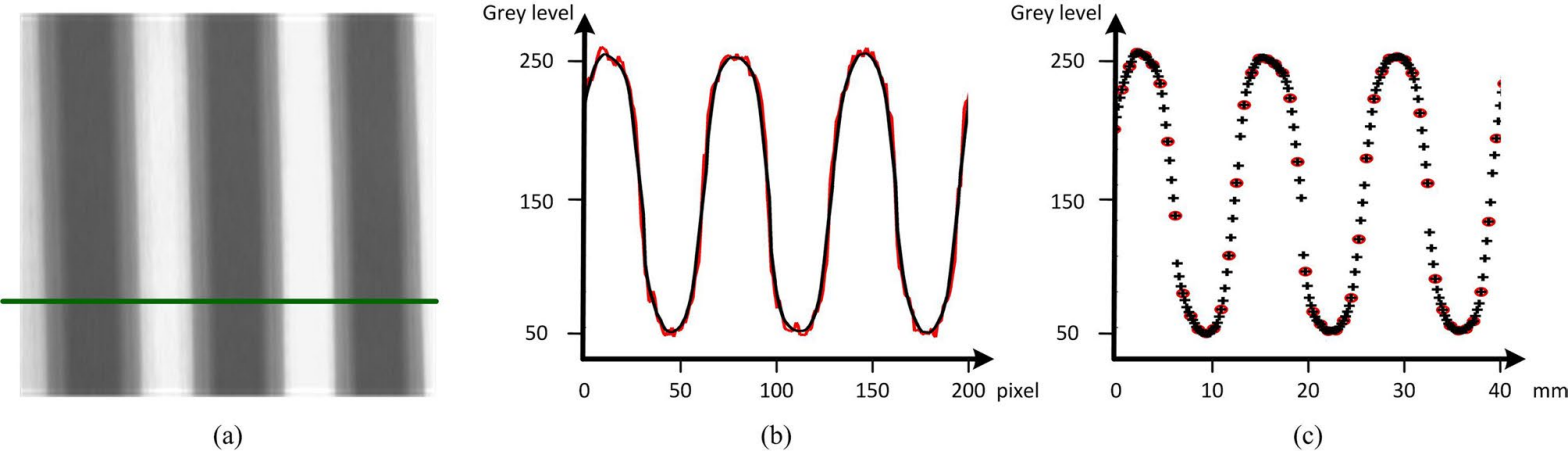
Comparative results. (**a**) The reconstructed stripe image. (**b**) Intensity distributions of the projected sinusoidal stripe in the reconstructed image of DMD camera and the image of traditional camera with N times resolution. (**c**) Spatial sampling distribution of the projected sinusoidal stripe in the reconstructed image and the non-reconstructed image.

We project the sinusoidal stripe and reconstruct it by applying CS theory on DMD camera. Figure 4 confirms the noise robustness and the enhancement of spatial sampling frequency. Figure 4a is the reconstructed sinusoidal stripe image. Green solid line is the cross section in the x direction. Black line in Fig. 4b is the intensity distributions of the projected sinusoidal stripe in the reconstructed image of DMD camera. Red line in Fig. 4b is the intensity distributions of the projected sinusoidal stripe in the traditional image with *N* times resolution respectively. The variable reflectivity and the multiple-reflection result in the sinusoidal stripe distortion which can be regarded as the random noise. The comparison shows that CS-based imaging method removes the noise effectively. Black ‘ + ’ and red ‘o’ in Fig. 4c are the spatial samples in reconstructed image and the non-reconstructed image respectively. The reconstructed image has *N* times as many resolutions as the previous image. Obviously, DMD camera can support the high-resolution imaging by mapping one pixel to multiple mirrors. Merged with CS theory, it can capture sinusoidal stripe image with low noise to improve the quality of phase solution. In a word, proposed CS-based imaging method has advantage in 3D shape measurement for the steep surface.

In this research, four-step phase shifting algorithm is employed to measure the steep surface. The DMD pattern sequence of four projections is identical to ensure the calculation of wrapped phase. The four reconstructed fringe images with step phase shift $\pi /2$ are expressed as

7 $$ \left\{ {\begin{array}{*{20}l} {\begin{array}{*{20}l} {\overset{\lower0.5em\hbox{$\smash{\scriptscriptstyle\frown}$}}{I}_{x,y}^{1} (n) = a_{x,y} + b_{x,y} \cos [\phi (\frac{n}{N}x,y)]} & {\begin{array}{*{20}l} {} & {} \\ \end{array} } \\ \end{array} } \\ {\overset{\lower0.5em\hbox{$\smash{\scriptscriptstyle\frown}$}}{I}_{x,y}^{2} (n) = a_{x,y} + b_{x,y} \cos [\phi (\frac{n}{N}x,y) + \pi /2]} \\ {\begin{array}{*{20}l} {\overset{\lower0.5em\hbox{$\smash{\scriptscriptstyle\frown}$}}{I}_{x,y}^{3} (n) = a_{x,y} + b_{x,y} \cos [\phi (\frac{n}{N}x,y) + \pi ]} & {} \\ \end{array} } \\ {\overset{\lower0.5em\hbox{$\smash{\scriptscriptstyle\frown}$}}{I}_{x,y}^{4} (n) = a_{x,y} + b_{x,y} \cos [\phi (\frac{n}{N}x,y) + 3\pi /2]} \\ \end{array} } \right.\quad {\text{or}}\quad \left\{ {\begin{array}{*{20}l} {\begin{array}{*{20}l} {\overset{\lower0.5em\hbox{$\smash{\scriptscriptstyle\frown}$}}{I}_{x,y}^{1} (n) = a_{x,y} + b_{x,y} \cos [\phi (x,\frac{n}{N}y)]} & {\begin{array}{*{20}l} {} & {} \\ \end{array} } \\ \end{array} } \\ {\overset{\lower0.5em\hbox{$\smash{\scriptscriptstyle\frown}$}}{I}_{x,y}^{2} (n) = a_{x,y} + b_{x,y} \cos [\phi (x,\frac{n}{N}y) + \pi /2]} \\ {\begin{array}{*{20}l} {\overset{\lower0.5em\hbox{$\smash{\scriptscriptstyle\frown}$}}{I}_{x,y}^{3} (n) = a_{x,y} + b_{x,y} \cos [\phi (x,\frac{n}{N}y) + \pi ]} & {} \\ \end{array} } \\ {\overset{\lower0.5em\hbox{$\smash{\scriptscriptstyle\frown}$}}{I}_{x,y}^{4} (n) = a_{x,y} + b_{x,y} \cos [\phi (x,\frac{n}{N}y) + 3\pi /2]} \\ \end{array} } \right. $$

where $a_{x,y}$ is the background intensity, and $b_{x,y}$ is the intensity amplitude. Thus the refined phase on the $n$ th sub-pixel of point $(x,y)$ is calculated by

8 $$ \begin{gathered} \begin{array}{*{20}l} {\phi (\frac{n}{N}x,y) = \arctan \left[ {\left( {\overset{\lower0.5em\hbox{$\smash{\scriptscriptstyle\frown}$}}{I}_{x,y}^{2} (n) - \overset{\lower0.5em\hbox{$\smash{\scriptscriptstyle\frown}$}}{I}_{x,y}^{4} (n)} \right)/\left( {\overset{\lower0.5em\hbox{$\smash{\scriptscriptstyle\frown}$}}{I}_{x,y}^{1} (n) - \overset{\lower0.5em\hbox{$\smash{\scriptscriptstyle\frown}$}}{I}_{x,y}^{3} (n)} \right)} \right]} & {{\text{or}}} \\ \end{array} \hfill \\ \phi (x,\frac{n}{N}y) = \arctan \left[ {\left( {\overset{\lower0.5em\hbox{$\smash{\scriptscriptstyle\frown}$}}{I}_{x,y}^{2} (n) - \overset{\lower0.5em\hbox{$\smash{\scriptscriptstyle\frown}$}}{I}_{x,y}^{4} (n)} \right)/\left( {\overset{\lower0.5em\hbox{$\smash{\scriptscriptstyle\frown}$}}{I}_{x,y}^{1} (n) - \overset{\lower0.5em\hbox{$\smash{\scriptscriptstyle\frown}$}}{I}_{x,y}^{3} (n)} \right)} \right] \hfill \\ \end{gathered} $$

where $\phi (\frac{n}{N}x,y)$ and $\phi (x,\frac{n}{N}y)$ are a wrapped phase ranging from –π to π. To measure the depth information, it is necessary to unwrap the phase. In this paper, two-frequency temporal phase unwrapping algorithm is employed to obtain the unwrapped phase as follows:

9 $$ \tilde{\phi }_{x,y} (n) = Mod[\phi_{x,y}^{l + 1} (n) - \phi_{x,y}^{l} (n),2\pi ] $$

10 $$ k_{x,y} (n) = Round\{ [l\tilde{\phi }_{x,y} (n) - \phi_{x,y}^{l} (n) - \pi ]/2\pi \} $$

11 $$ \overline{\phi }_{x,y} (n) = \phi_{x,y}^{l} (n) + 2\pi \cdot k_{x,y} (n) + \pi $$

where $\tilde{\phi }_{x,y} (n)$ is the normalized phase, $\phi_{x,y}^{l} (n)$ and $\phi_{x,y}^{l + 1} (n)$ are the wrapped phase with fringe frequency $P/l$ and $P/(l + 1)$ respectively, $P$ is the normalized period. Function $Mod[ \bullet ]$ is to obtain the remainder when $\phi_{x,y}^{l + 1} (n) - \phi_{x,y}^{l} (n)$ is divided by 2π. $k_{x,y} (n)$ is the sequence number of the unwrapped phase period, function $Round[ \bullet ]$ is to get the nearest integer. To reduce the phase error and enhance the signal noise ratio (SNR) of the reconstructed form, median filtering is usually performed after Eq. (10). $\overline{\phi }_{x,y} (n)$ is the ultimate unwrapped phase ranging from 0 to 2kπ. The unwrapped phase will be converted to 3D coordinate by the phase-to-depth map. Based on above proposed method, three-dimensional shape measurement for the steep surface can be implemented.

## Experiments

**Figure 5:**
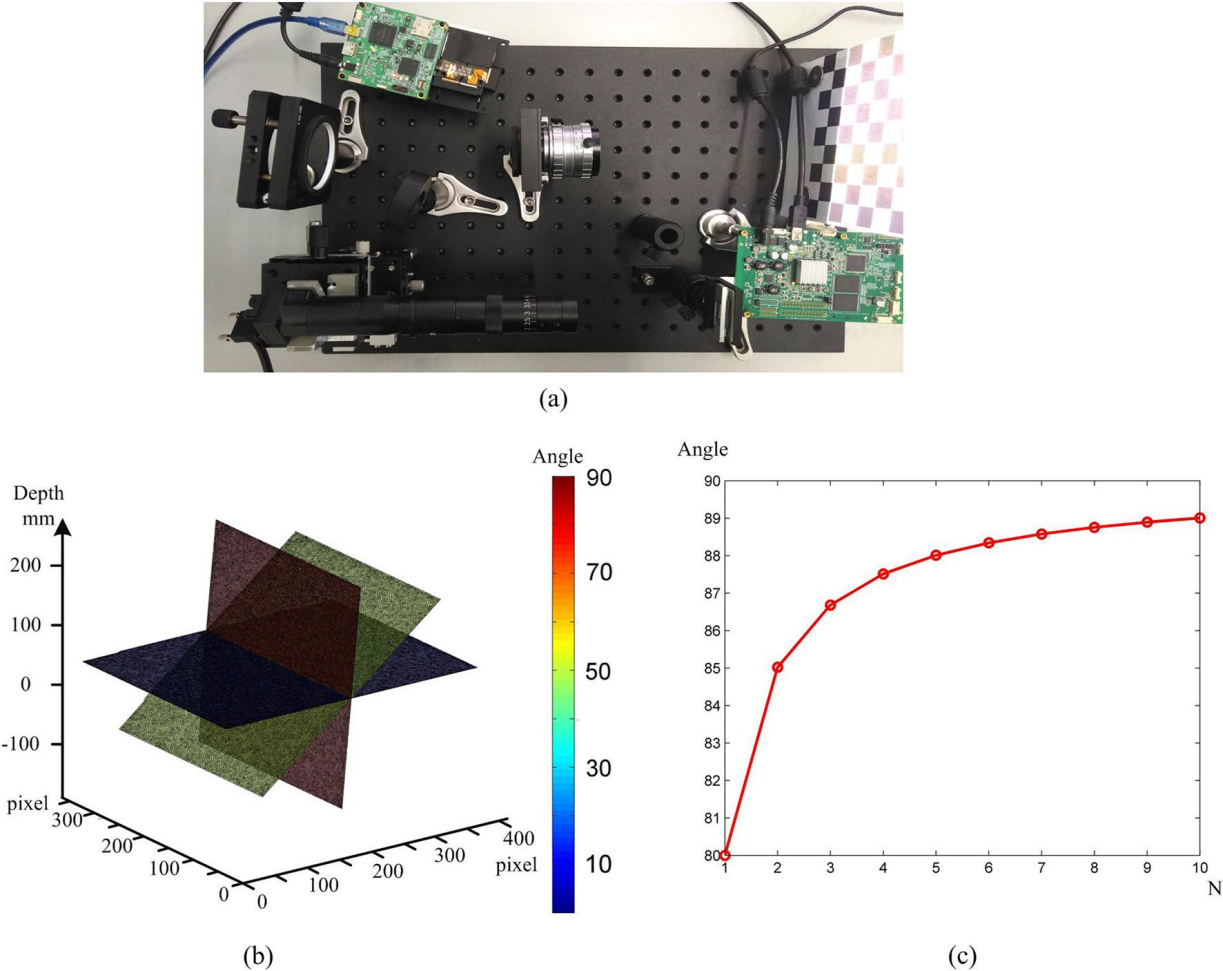
The experimental results of measuring planes with various angles. (**a**) The experimental setup. (**b**) 3D reconstructed planes with various angles. (**c**) Maximum measurable angle with different parameter N.

To assess the ability to measure steep surface, we took the planes with various slopes as objects in 3D measurement. Figure 5 is the results that the planes with various slopes are measured by using our proposed method. Figure 5a is the experimental setup. The different color planes reconstructed in Fig. 5b refer to the surfaces with various slopes. The depth direction is identical to the primary optical axis of the objective lens. Figure 5c demonstrates the correlation that maximum measurable angle is gradually enhanced with the increasement of parameter *N*. Under the constraint of root mean square (RMS), the maximum measurable angle $$\overset{\lower0.5em\hbox{$\smash{\scriptscriptstyle\frown}$}}{\alpha }$$ of sloping plane which is fitted by least square function approximation can be expressed by:

12 $$ \overset{\lower0.5em\hbox{$\smash{\scriptscriptstyle\frown}$}}{\alpha }_{x,y} = \arg \max \left\| {\alpha_{x,y} } \right\|_{0} \quad {\text{s.t}}\quad \left\| {{\mathbb{Z}}_{x,y} - \overset{\lower0.5em\hbox{$\smash{\scriptscriptstyle\frown}$}}{\mathbb{Z}}_{x,y} } \right\|_{2} < \varepsilon $$

where $${\mathbb{Z}}_{x,y}$$ is the measured depth value at point (x, y), and $$\overset{\lower0.5em\hbox{$\smash{\scriptscriptstyle\frown}$}}{\mathbb{Z}}_{x,y}$$ denotes the least squares approximating value. As Fig. 5 indicates, we can obtain that parameter *N* is determined by calculating the slope angle of measured surface on the following measurement. Table 1 shows the errors of the reconstructed plane with the different *N*. The measurement speed is mainly determined by the number of captured images. The measurement accuracy depends on the spatial resolution. Both of them are related with *N*. Considering the tradeoff between the captured times and reconstructed accuracy, *N* is selected from 2 to 5. The angle of the reconstructed sloping plane is 85 degrees. By fitting these planar point clouds using the least-squares method, the maximum surplus error *E*_m_ is used to verify the reconstructed accuracy on the edge. The standard deviation *σ* illustrates that our method efficiently decreases the noise of the steep surface.

**Table 1:** The errors of the reconstructed plane with the different N.

*N*	2	3	4	5
*E*_m_ (mm)	0.20	0.07	0.06	0.05
*σ*(mm)	0.08	0.04	0.02	0.02

**Figure 6:**
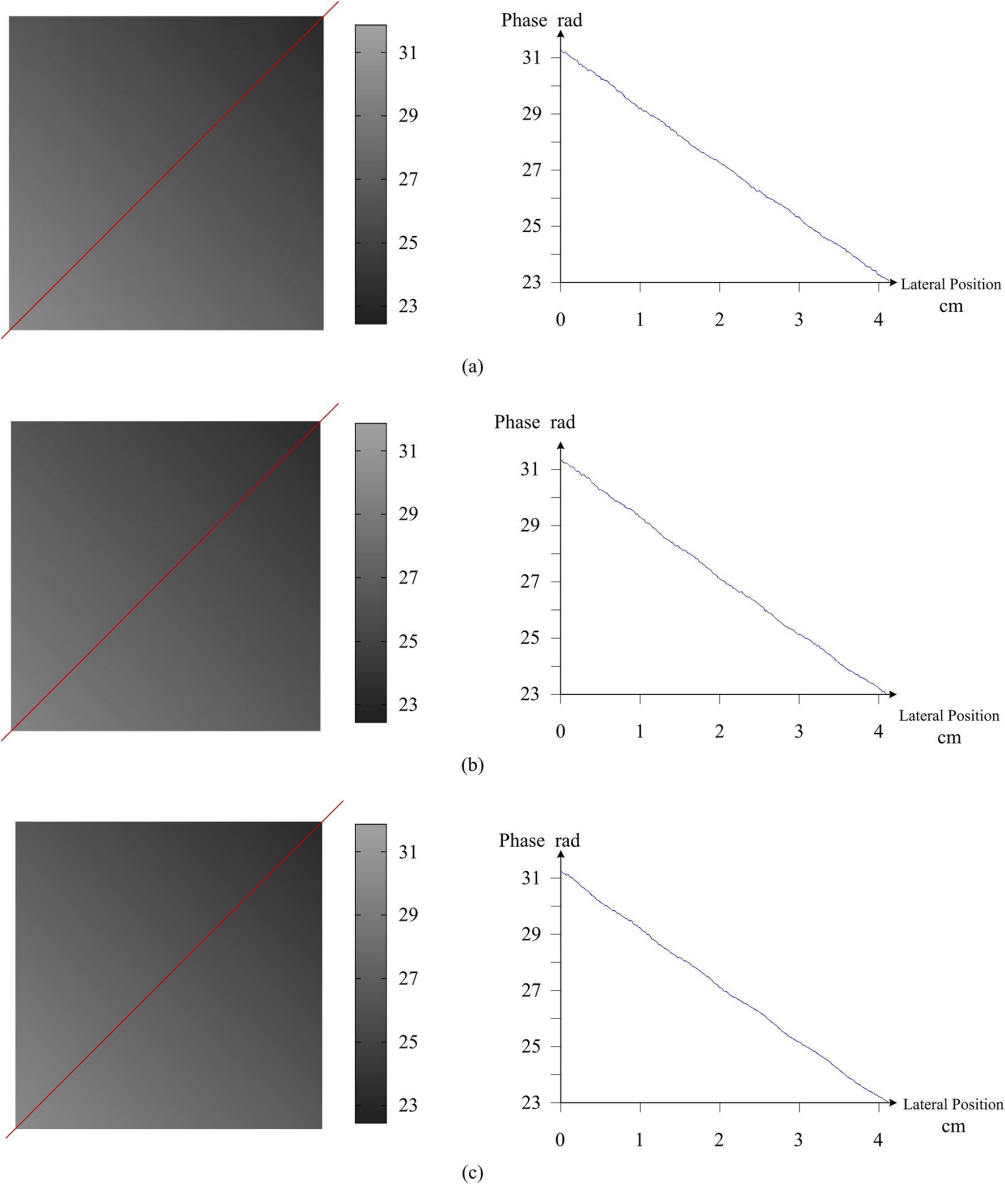
The cross section of phase map in the measurement experiment of the same plane. (**a**) obtained by Zhan Song’s method. (**b**) obtained by Rosario’s method. (**c**) obtained by Proposed method.

Figure 6 is the phase distribution of the measured plane obtained by different methods. For comparison, we choose Zhan Song’s method that the gridline pattern is designed to encode localization with sub-pixel accuracy and Rosario’s method that generates a series of projection patterns with a minimal gradient in the intensity to reduce errors near the border^7,8^. The mirror number of 5 × 5 is used in our method. Red diagonal is the cross section of phase map. To align diamond-shaped DMD mirror, camera has been rotated 45 degrees clockwise. The errors of reconstructed plane with different methods are shown in Table 2. *E*_a_ denotes the difference between the ground truth and the calculated position of fitted plane. These results indicate that proposed method has higher accuracy than other methods.

**Table 2:** The errors of the reconstructed plane with different methods.

Methods	*E*_a_ (mm)	*E*_m_ (mm)	*σ*(mm)
Zhan Song’s method	0.12	0.24	0.06
Rosario’s method	0.17	0.31	0.03
Proposed method	0.04	0.05	0.02

**Figure 7:**
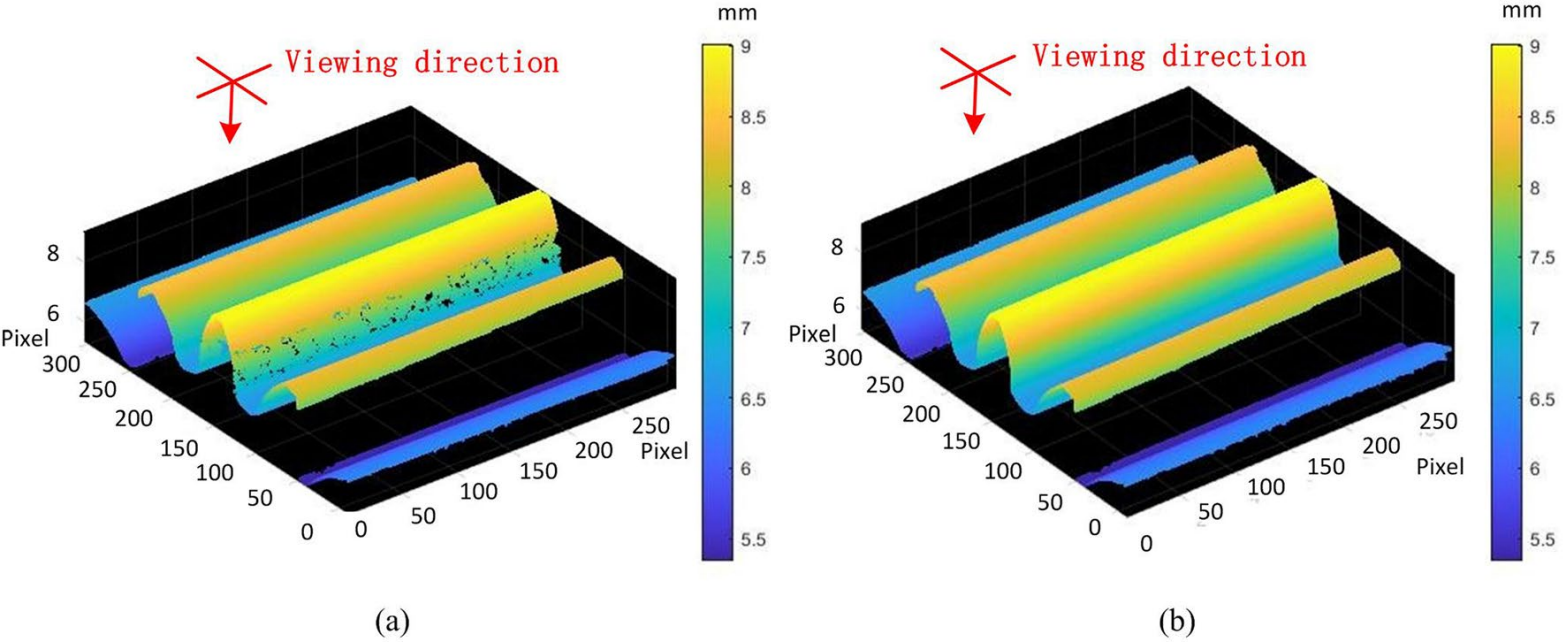
The comparison between the reconstructed gear teeth (**a**) with traditional four-step PSP and the reconstructed gear teeth (**b**) with proposed method.

3D shape measurement for the involute gear is an appropriate application of proposed method. Since the tooth profile and the tooth root fillet determine the contact force when one gear is driving another gear, the force analysis demands to measure them accurately. To avoid the occlusion from neighbor teeth, we used the proposed method to measure the tooth profile and the tooth root fillet in Fig. 7b. Figure 7a is the measuring result of the gear teeth with traditional four-step phase shifting profilometry (PSP). The defects on the tooth profile and the tooth root fillet would result in the failure to analyze the force in Fig. 7a. The measuring result in Fig. 7b shows that our proposed method can eliminate those defects.

**Figure 8:**
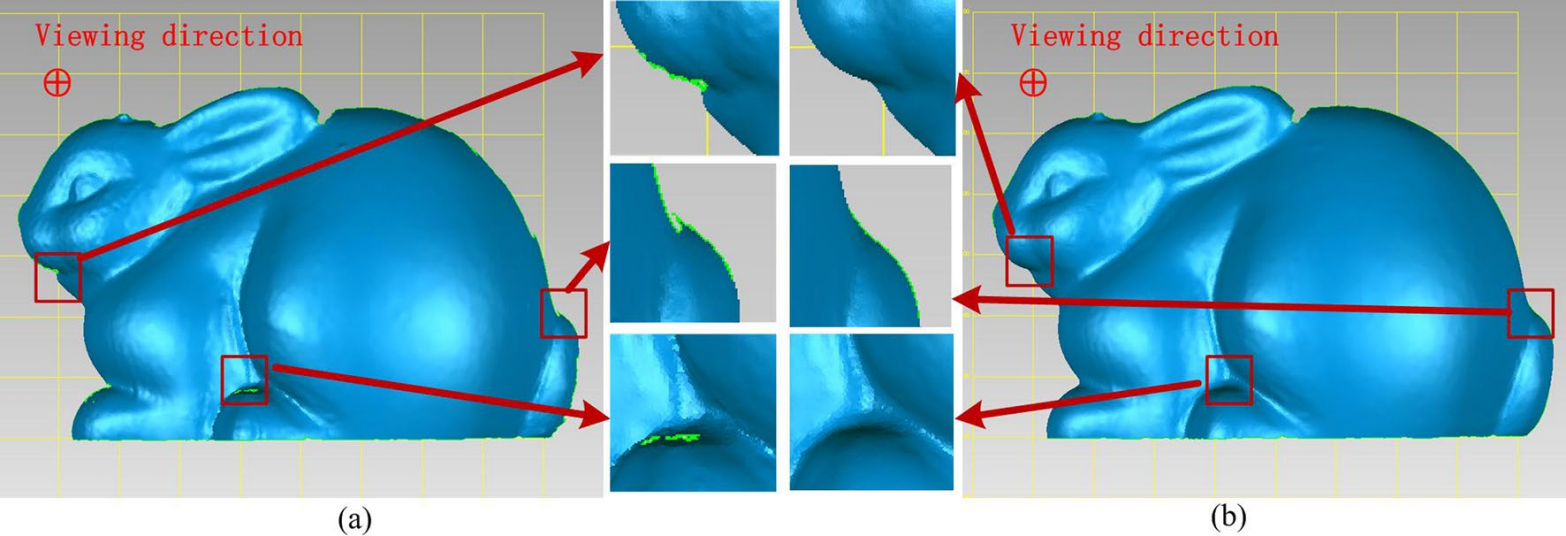
The comparison between the reconstructed rabbit (**a**) with traditional four-step PSP and the reconstructed rabbit (**b**) with proposed method.

We measured the rabbit model to verify the performance of proposed method in Fig. 8 too. There are many gaps on the edge of the reconstructed rabbit in Fig. 8a. It is difficult to reconstruct the acute characteristics of plaster model. Our method enhances the spatial resolution to improve the reconstructed performance on the acute angle edge. The reconstructed edge becomes plump in Fig. 8b. The comparative results in Figs. 7 and 8 indicate that our proposed method can improve 3D shape measurement for the steep surface to a certain extent.

Four-step phase shifting profilometry requires 4 projecting patterns. To meet the two-frequency temporal phase unwrapping algorithm and four-step phase shifting profilometry, projector will give 8 sinusoidal stripes. For a complete measurement based on our method, 8* M* captured times will be taken, $M \le N$. The number *N* determines the time consumption besides the reconstructed quality of CS, as shown in Table 3. In our experiment, CR (compressive rate) is 0.6 at least, e.g., if N was 5, M would be 3. Totally, camera will capture 24 images to complete one measurement.

**Table 3:** The captured times with the different N.

	Projecting pattern number	Capture times 4 M
N = 3	2 × 4	16 (CR = 0.67)
N = 4	2 × 4	24 (CR = 0.75)
N = 5	2 × 4	24 (CR = 0.6) or 32(CR = 0.8)

## Conclusion

In this works, we presented the method that CS theory is used to capture phase shifting images in DMD camera to measure steep surface. Specially, DMD camera system that one CCD pixel can be assigned to multiple DMD mirrors was constructed. Two directional measurement matrices from the same orthogonal square matrix were used to modulate the DMD segments by judging the gradient direction of original image. Thus the sinusoidal stripe images with phase shifting π/2 were reconstructed to obtain the three-dimensional shape of the measured object. Finally, we measured the involute gear and the rabbit model to demonstrate that 3D shape measurement for the steep surface can be improved by our proposed method. It is obvious that the method in this paper has advantages in the noise robustness and the enhancement of spatial sampling frequency. However, the quality of reconstructed phase shifting images is limited by the compression ratio. To trade off the reconstructed quality against the time consumption, we did not adopt the measurement matrix with the dimension $M \times N^{2}$ in classical CS theory. Furthermore, our method is able to reconstruct the three dimensional shape of the obtuse angle edge. The acute angle edge is difficult to measure due to the ambiguity of the edge. Our proposed method can be a compensation for other phase shifting profilometry technology, e.g., this method can merge with dual-camera PSP technology to enhance the measurement accuracy of steep surface further. Our group will focus the research on DMD scanning alignment method which would break through the limitation of DMD resolution in the future.

## Data Availability

The datasets generated during and/or analyzed during the current study are available from the corresponding author on reasonable request.

## References

[1] Geng J (2011). Structured-light 3D surface imaging: a tutorial. Adv. Opt. Photon..

[2] Guehring J (2000). Dense 3D surface acquisition by structured light using off-the-shelf components. Proc. Photonics West 2001 Electronic Imaging..

[3] Salvi J, Fernandez S, Pribanic T, Llado X (2010). A state of the art in structured light patterns for surface profilometry. Pattern Recogn..

[4] Wang S, Zhu Z, Zhang H, Zhou F (2020). Suppression method for strong interference from stray light in 3D imaging system of structured light. Opt. Eng..

[5] Yu C, Ji F, Jing X, Liu M (2019). Dynamic granularity matrix space based adaptive edge detection method for structured light stripes. Math. Probl. Eng..

[6] Wang Y, Liu K, Hao Q, Lau DL, Hassebrook LG (2011). Period coded phase shifting strategy for real–time 3-D structured light illumination. IEEE Trans. Image Process..

[7] Song Z, Tang S, Gu F, Shi C, Feng J (2019). DOE-based structured-light method for accurate 3D sensing. Opt. Lasers Eng..

[8] Porras-Aguilar R, Falaggis K, Ramos-Garcia R (2017). Optimum projection pattern generation for grey-level coded structured light illumination systems. Opt. Lasers Eng..

[9] Jia X, Zhang Z, Cao F, Zeng D (2010). Model and error analysis for coded structured light measurement system. Opt. Eng..

[10] Danzl R, Helmli F, Scherer S (2011). Focus variation - a robust technology for high resolution optical 3D surface metrology. Stroj Vestn-J. Mech. E.

[11] Newton L (2019). Areal topography measurement of metal additive surfaces using focus variation microscopy. Addit. Manuf..

[12] Otani, Y., Kobayashi, F., Mizutani, Y., & Yoshizawa, T. Three-dimensional profilometry based on focus method by projecting grating pattern. *Proc. Opt. Inspect. Metrol. Non-Opt. Indust.* **7432**, 278–283 (2009).

[13] Okatani IS, Deguchi K (2002). A method for fine registration of multiple view range images considering the measurement error properties. Comput. Vis. Image Underst..

[14] Qian J, Tao T, Feng S, Chen Q, Zuo C (2019). Motion-artifact-free dynamic 3D shape measurement with hybrid Fourier-transform phase-shifting profilometry. Opt. Express.

[15] Feng SJ, Chen Q, Zuo C, Asundi A (2017). Fast three-dimensional measurements for dynamic scenes with shiny surfaces. Opt. Commun..

[16] Jiang CF, Zhang S (2017). Absolute phase unwrapping for dual-camera system without embedding statistical features. Opt. Eng..

[17] Shi, W. Z. et al. Cardiac image super-resolution with global correspondence using multi-atlas PatchMatch. *Proc. Medical Image Comput. Comput. Assist. Intervent.* **8151**, 9–16. 10.1007/978-3-642-40760-4_2 (2013). 10.1007/978-3-642-40760-4_2 24505738

[18] Yang, Q., Yang, R., Davis, J., Nister, D. & Ieee. Spatial-depth super resolution for range images. *Proc. Comput. Vis. Pattern Recognit.* **2007**, 1–8. 10.1109/CVPR.2007.383211 (2007).

[19] Walecki P, Taubin G (2020). Super-resolution 3-D laser scanning based on interval arithmetic. IEEE Trans. Instrum. Meas..

[20] Donoho DL (2006). Compressed sensing. IEEE Trans. Inf. Theory.

[21] Candes EJ, Wakin MB (2008). An introduction to compressive sampling. IEEE Signal Process. Mag..

[22] Massa A, Rocca P, Oliveri G (2015). Compressive sensing in electromagnetics-a review. IEEE Antennas Propag. Mag..

[23] Zhang Y (2015). Exponential wavelet iterative shrinkage thresholding algorithm for compressed sensing magnetic resonance imaging. Inf. Sci..

[24] Kovarik L, Stevens A, Liyu A, Browning ND (2016). Implementing an accurate and rapid sparse sampling approach for low-dose atomic resolution STEM imaging. Appl. Phys. Lett..

[25] Duarte MF (2008). Single-pixel imaging via compressive sampling. IEEE Signal Process. Mag..

[26] Edgar MP, Gibson GM, Padgett MJ (2019). Principles and prospects for single-pixel imaging. Nat. Photonics.

[27] Sun M-J, Edgar MP, Phillips DB, Gibson GM, Padgett MJ (2016). Improving the signal-to-noise ratio of single-pixel imaging using digital microscanning. Opt. Express.

[28] Magalhaes F, Araujo FM, Correia MV, Abolbashari M, Farahi F (2011). Active illumination single-pixel camera based on compressive sensing. Appl. Opt..

[29] Fujigaki M, Morimoto Y, Gao Q (2002). Shape and displacement measurement by phase-shifting scanning moire method using digital micromirror device. Proc. Third Int. Conf. Exp. Mech..

[30] Zhao S-B, Zhang F-M, Qu X-H, Chen Z, Zheng S-W (2014). Removal of parasitic image due to metal specularity based on digital micromirror device camera. Opt. Eng..

[31] Zhao S-B, Liu L-Y, Ma M-Y (2019). Adaptive high-dynamic range three-dimensional shape measurement using DMD camera. Ieee Access.

[32] Zhao SB, Ma MY, Guo C (2018). Accurate pixel-to-pixel alignment method with six-axis adjustment for computational photography. IEEE Photonics J..

[33] Sahoo SK, Makur A (2015). Signal recovery from random measurements via extended orthogonal matching pursuit. IEEE Trans. Signal Process..

